# Supplementary material for: Parental views on their children’s smartphone use during personal and relational activities
Source: PLoS One. 2024 Aug 5;19(8):e0308258. doi: 10.1371/journal.pone.0308258 (PMC11299814; doi:10.1371/journal.pone.0308258)
Supplement: S1 Table — (DOCX) [file pone.0308258.s001.docx]

**Table S1.** Characteristics of participants.

|  | | Whole sample (n = 826) | |  | Children 6-10 years (N = 423) | |  | Adolescents 11-18 years (N = 403) | |
| --- | --- | --- | --- | --- | --- | --- | --- | --- | --- |
|  |  | n | % |  | N | % |  | N | % |
| Parent gender | |  |  |  |  |  |  |  |  |
|  | Female | 408 | 49.4 |  | 223 | 52.7 |  | 185 | 45.9 |
|  | Male | 418 | 50.6 |  | 200 | 47.3 |  | 218 | 54.1 |
|  |  |  |  |  |  |  |  |  |  |
| Parent age – Mean (Standard deviation) in years | | 43.5 (4.40) | |  | 40.7 (4.10) | |  | 46.4 (4.72) | |
|  | 25-34 years | 90 | 10.9 |  | 76 | 18.0 |  | 14 | 3.5 |
|  | 35-44 years | 379 | 45.9 |  | 250 | 59.1 |  | 129 | 32.0 |
|  | 45-54 years | 302 | 36.6 |  | 82 | 19.4 |  | 220 | 54.6 |
|  | 55-64 years | 45 | 5.4 |  | 10 | 2.3 |  | 35 | 8.7 |
|  | More than 65 years | 10 | 1.2 |  | 5 | 1.2 |  | 5 | 1.2 |
|  |  |  |  |  |  |  |  |  |  |
| Parent education | |  |  |  |  |  |  |  |  |
|  | Elementary or lower | 16 | 1.9 |  | 10 | 2.4 |  | 6 | 1.5 |
|  | Practical | 123 | 14.9 |  | 56 | 13.2 |  | 67 | 16.6 |
|  | High school with graduation | 412 | 49.9 |  | 207 | 48.9 |  | 205 | 50.9 |
|  | University or higher | 275 | 33.3 |  | 150 | 35.5 |  | 125 | 31.0 |
|  |  |  |  |  |  |  |  |  |  |
| The size of residential area | |  |  |  |  |  |  |  |  |
|  | Less than 999 inhabitants | 103 | 12.5 |  | 55 | 13.0 |  | 48 | 11.9 |
|  | 1 000-4 999 inhabitants | 127 | 15.4 |  | 68 | 16.1 |  | 59 | 14.6 |
|  | 5 000-19 999 inhabitants | 153 | 18.5 |  | 82 | 19.4 |  | 71 | 17.6 |
|  | 20 000-99 999 inhabitants | 211 | 25.5 |  | 106 | 25.0 |  | 105 | 26.1 |
|  | More than 100 000 inhabitants | 232 | 28.1 |  | 112 | 26.5 |  | 120 | 29.8 |
|  |  |  |  |  |  |  |  |  |  |
| Household income | |  |  |  |  |  |  |  |  |
|  | Less than 800 EUR | 36 | 4.4 |  | 18 | 4.2 |  | 18 | 4.5 |
|  | 801-1600 EUR | 196 | 23.7 |  | 101 | 23.9 |  | 95 | 23.6 |
|  | More than 1600 CZK | 535 | 64.8 |  | 272 | 64.3 |  | 263 | 65.2 |
|  | No response | 59 | 7.1 |  | 32 | 7.6 |  | 27 | 6.7 |
|  |  |  |  |  |  |  |  |  |  |
| Socioeconomic status | |  |  |  |  |  |  |  |  |
|  | Very high (A) | 113 | 13.7 |  | 55 | 13.0 |  | 58 | 14.4 |
|  | High (B) | 133 | 16.1 |  | 62 | 14.7 |  | 71 | 17.6 |
|  | Moderate – high (C1) | 142 | 17.2 |  | 70 | 16.5 |  | 72 | 17.9 |
|  | Moderate – average (C2) | 135 | 16.3 |  | 68 | 16.1 |  | 67 | 16.6 |
|  | Moderate – low (C3) | 139 | 16.8 |  | 71 | 16.8 |  | 68 | 16.9 |
|  | Low or very low (D1, D2, E) | 164 | 19.9 |  | 97 | 22.9 |  | 67 | 16.6 |
|  |  |  |  |  |  |  |  |  |  |
| Size of household (the number of children living in the household) | |  |  |  |  |  |  |  |  |
|  | 1 child | 466 | 56.4 |  | 201 | 47.5 |  | 265 | 65.7 |
|  | 2 children | 308 | 37.3 |  | 186 | 44.0 |  | 122 | 30.3 |
|  | 3 or more children | 52 | 6.3 |  | 36 | 8.5 |  | 16 | 4.0 |
|  |  |  |  |  |  |  |  |  |  |
| Family intactness | |  |  |  |  |  |  |  |  |
|  | Intact family (child lives with both parents) | 565 | 68.4 |  | 315 | 74.5 |  | 250 | 62.0 |
|  | Single parent family | 118 | 14.3 |  | 39 | 9.2 |  | 79 | 19.6 |
|  | Completed family (child lives with one parent and his or her new partner) | 87 | 10.5 |  | 41 | 9.7 |  | 46 | 11.4 |
|  | Shared custody | 48 | 5.8 |  | 22 | 5.2 |  | 26 | 6.5 |
|  | Other | 8 | 1.0 |  | 6 | 1.4 |  | 2 | 0.5 |
|  |  |  |  |  |  |  |  |  |  |
| Child gender | |  |  |  |  |  |  |  |  |
|  | Female | 401 | 48.5 |  | 221 | 52.2 |  | 180 | 44.7 |
|  | Male | 425 | 51.5 |  | 202 | 47.8 |  | 223 | 55.3 |
|  |  |  |  |  |  |  |  |  |  |
| Child’s position among siblings | |  |  |  |  |  |  |  |  |
|  | The only child | 157 | 19.0 |  | 68 | 16.1 |  | 89 | 22.1 |
|  | Has only younger sibling/s | 172 | 20.8 |  | 130 | 30.7 |  | 42 | 10.4 |
|  | Has only older sibling/s | 409 | 49.5 |  | 185 | 43.7 |  | 224 | 55.6 |
|  | Has both younger and older sibling/s | 71 | 8.6 |  | 35 | 8.3 |  | 36 | 8.9 |
|  | Has a twin | 17 | 2.1 |  | 5 | 1.2 |  | 12 | 3.0 |
|  |  |  |  |  |  |  |  |  |  |
| Child’s ownership of devices: | |  |  |  |  |  |  |  |  |
|  | Smartphone owner | 662 | 80.1 |  | 266 | 62.9 |  | 396 | 98.3 |
|  | Tablet owner | 311 | 37.7 |  | 169 | 40.0 |  | 142 | 35.2 |
|  | Gaming console owner | 184 | 22.3 |  | 67 | 15.8 |  | 117 | 29.0 |
|  | Computer owner | 417 | 50.5 |  | 120 | 28.4 |  | 297 | 73.7 |
|  | Television owner | 314 | 38.0 |  | 154 | 36.4 |  | 160 | 39.7 |
|  |  |  |  |  |  |  |  |  |  |
| Child’s age | |  |  |  |  |  |  |  |  |
|  | 6 years | 111 | 13.4 |  | - | - |  | - | - |
|  | 7 years | 89 | 10.8 |  | - | - |  | - | - |
|  | 8 years | 63 | 7.6 |  | - | - |  | - | - |
|  | 9 years | 92 | 11.1 |  | - | - |  | - | - |
|  | 10 years | 68 | 8.2 |  | - | - |  | - | - |
|  | 11 years | 58 | 7.0 |  | - | - |  | - | - |
|  | 12 years | 73 | 8.8 |  | - | - |  | - | - |
|  | 13 years | 70 | 8.5 |  | - | - |  | - | - |
|  | 14 years | 68 | 8.2 |  | - | - |  | - | - |
|  | 15 years | 51 | 6.2 |  | - | - |  | - | - |
|  | 16 years | 32 | 3.9 |  | - | - |  | - | - |
|  | 17 years | 36 | 4.4 |  | - | - |  | - | - |
|  | 18 years | 15 | 1.8 |  | - | - |  | - | - |
|  |  |  |  |  |  |  |  |  |  |
| Child’s school year | |  |  |  |  |  |  |  |  |
|  | Preschool | 48 | 5.8 |  | - | - |  | - | - |
|  | Primary school (Elementary grades 1-5) | 412 | 49.9 |  | - | - |  | - | - |
|  | Secondary school (Elementary grades 6-9) | 248 | 30.0 |  | - | - |  | - | - |
|  | High school or University | 118 | 14.3 |  | - | - |  | - | - |
