# Supplementary material for: Parental views on their children’s smartphone use during personal and relational activities
Source: PLoS One. 2024 Aug 5;19(8):e0308258. doi: 10.1371/journal.pone.0308258 (PMC11299814; doi:10.1371/journal.pone.0308258)
Supplement: S2 Table — Frequencies of responses and their proportions in percentages are displayed. (DOCX) [file pone.0308258.s002.docx]

**Table S2.** Parental views on children’s smartphone use during personal and relational activities in parents of children aged 6–10 years (N = 423). Frequencies of responses and their proportions in percentages are displayed.

|  | | Responses | | | | |  |
| --- | --- | --- | --- | --- | --- | --- | --- |
|  |  |  | | | | |  |
| How would you feel if your child would use a smartphone during following activity: | | I wouldn't mind it at all | I wouldn't mind it | Neutral | I would mind it | I couldn't stand it |  |
|  |  |  |  |  |  |  |  |
|  |  |  |  |  |  |  |  |
|  | During family mealtime | 11 | 31 | 34 | 74 | 273 |  |
|  |  | 2.6% | 7.3% | 8.0% | 17.5% | 64.5% |  |
|  |  |  |  |  |  |  |  |
|  | When visiting family friends | 10 | 49 | 43 | 145 | 176 |  |
|  |  | 2.4% | 11.6% | 10.2% | 34.3% | 41.6% |  |
|  |  |  |  |  |  |  |  |
|  | During dinning in the restaurant | 6 | 29 | 31 | 82 | 275 |  |
|  |  | 1.4% | 6.9% | 7.3% | 19.4% | 65.0% |  |
|  |  |  |  |  |  |  |  |
|  | When s/he is supposed to focus on something else (e.g., studying) | 3 | 11 | 18 | 46 | 345 |  |
|  |  | 0.7% | 2.6% | 4.3% | 10.9% | 81.6% |  |
|  |  |  |  |  |  |  |  |
|  | When walking or hiking or being on trip together | 15 | 37 | 33 | 96 | 242 |  |
|  |  | 3.5% | 8.7% | 7.8% | 22.7% | 57.2% |  |
|  |  |  |  |  |  |  |  |
|  | When travelling/commuting together | 69 | 152 | 49 | 83 | 70 |  |
|  |  | 16.3% | 35.9% | 11.6% | 19.6% | 16.5% |  |
|  |  |  |  |  |  |  |  |
|  | When you are saying something important to him or her | 4 | 11 | 22 | 56 | 330 |  |
|  |  | 0.9% | 2.6% | 5.2% | 13.2% | 78.0% |  |
|  |  |  |  |  |  |  |  |
|  | During parent‒child conversation | 7 | 19 | 34 | 96 | 267 |  |
|  |  | 1.7% | 4.5% | 8.0% | 22.7% | 63.1% |  |
|  |  |  |  |  |  |  |  |
|  | During a conversation with his or her peer (e.g., sibling, friend etc.) | 9 | 44 | 63 | 147 | 160 |  |
|  |  | 2.1% | 10.4% | 14.9% | 34.8% | 37.8% |  |
|  |  |  |  |  |  |  |  |
|  | When playing with a peer (e.g., sibling, friend etc.) | 12 | 53 | 57 | 141 | 160 |  |
|  |  | 2.8% | 12.5% | 13.5% | 33.3% | 37.8% |  |
|  |  |  |  |  |  |  |  |
|  | When attending a cultural performance (e.g., theatre, cinema, concert) | 5 | 10 | 18 | 35 | 355 |  |
|  |  | 1.2% | 2.4% | 4.3% | 8.3% | 83.9% |  |
|  |  |  |  |  |  |  |  |
|  |  |  |  |  |  |  |  |
|  |  |  |  |  |  |  |  |
|  | When a child is supposed to be focusing on something else (e.g., studying) | 3 | 11 | 18 | 46 | 345 |  |
|  |  | 0.7% | 2.6% | 4.3% | 10.9% | 81.6% |  |
|  |  |  |  |  |  |  |  |
|  | During bedtime | 7 | 16 | 19 | 58 | 323 |  |
|  |  | 1.7% | 3.8% | 4.5% | 13.7% | 76.4% |  |
|  |  |  |  |  |  |  |  |
|  |  |  |  |  |  |  |  |
|  | While waiting (e.g., at bus stop) | 127 | 175 | 37 | 56 | 28 |  |
|  |  | 30.0% | 41.4% | 8.7% | 13.2% | 6.6% |  |
|  |  |  |  |  |  |  |  |
|  | In the bathroom (toilet) | 50 | 99 | 83 | 103 | 88 |  |
|  |  | 11.8% | 23.4% | 19.6% | 24.3% | 20.8% |  |
