# Supplementary material for: Parental views on their children’s smartphone use during personal and relational activities
Source: PLoS One. 2024 Aug 5;19(8):e0308258. doi: 10.1371/journal.pone.0308258 (PMC11299814; doi:10.1371/journal.pone.0308258)
Supplement: S3 Table — Frequencies of responses and their proportions in percentages are displayed. (DOCX) [file pone.0308258.s003.docx]

**Table S3.** Parental views on children’s smartphone use during personal and relational activities in parents of adolescents aged 11–18 years (N = 403). Frequencies of responses and their proportions in percentages are displayed.

|  | | Responses | | | | | | | | | |  | |  |
| --- | --- | --- | --- | --- | --- | --- | --- | --- | --- | --- | --- | --- | --- | --- |
|  |  |  | | | | | | | | | |  | |  |
| How would you feel if your child would use a smartphone during following activity: | | I wouldn't mind it at all | | I wouldn't mind it | | Neutral | | I would mind it | | I couldn't stand it | |  | |  |
|  | During family mealtime | | 10 | | 35 | | 22 | | 82 | | 254 | |  | |
|  |  |  | 2.5% | | 8.7% | | 5.5% | | 20.3% | | 63.0% | |  | |
|  |  | |  | |  | |  | |  | |  | |  | |
|  | When visiting family friends | | 6 | | 51 | | 54 | | 138 | | 154 | |  | |
|  |  |  | 1.5% | | 12.7% | | 13.4% | | 34.2% | | 38.2% | |  | |
|  |  | |  | |  | |  | |  | |  | |  | |
|  | During dinning in the restaurant | | 5 | | 39 | | 31 | | 91 | | 237 | |  | |
|  |  |  | 1.2% | | 9.7% | | 7.7% | | 22.6% | | 58.8% | |  | |
|  |  | |  | |  | |  | |  | |  | |  | |
|  | When walking or hiking or being on trip together | | 16 | | 53 | | 65 | | 125 | | 144 | |  | |
|  |  |  | 4.0% | | 13.2% | | 16.1% | | 31.0% | | 35.7% | |  | |
|  |  | |  | |  | |  | |  | |  | |  | |
|  | When travelling/commuting together | | 74 | | 139 | | 49 | | 93 | | 48 | |  | |
|  |  | | 18.4% | | 34.5% | | 12.2% | | 23.1% | | 11.9% | |  | |
|  |  | |  | |  | |  | |  | |  | |  | |
|  | When you are saying something important to your child | | 4 | | 18 | | 30 | | 55 | | 296 | |  | |
|  |  |  | 1.0% | | 4.5% | | 7.4% | | 13.6% | | 73.4% | |  | |
|  |  | |  | |  | |  | |  | |  | |  | |
|  | During parent‒child conversation | | 3 | | 38 | | 34 | | 95 | | 233 | |  | |
|  |  |  | 0.7% | | 9.4% | | 8.4% | | 23.6% | | 57.8% | |  | |
|  |  | |  | |  | |  | |  | |  | |  | |
|  | During a conversation with his or her peer (e.g., sibling, friend etc.) | | 6 | | 57 | | 92 | | 135 | | 113 | |  | |
|  |  |  | 1.5% | | 14.1% | | 22.8% | | 33.5% | | 28.0% | |  | |
|  |  | |  | |  | |  | |  | |  | |  | |
|  | When playing with a peer (e.g., sibling, friend etc.) | | 8 | | 51 | | 79 | | 149 | | 116 | |  | |
|  |  |  | 2.0% | | 12.7% | | 19.6% | | 37.0% | | 28.8% | |  | |
|  |  | |  | |  | |  | |  | |  | |  | |
|  | When attending a cultural performance (e.g., theatre, cinema, concert) | | 3 | | 16 | | 27 | | 34 | | 323 | |  | |
|  |  |  | 0.7% | | 4.0% | | 6.7% | | 8.4% | | 80.1% | |  | |
|  |  | |  | |  | |  | |  | |  | |  | |
|  |  | |  | |  | |  | |  | |  | |  | |
|  |  | |  | |  | |  | |  | |  | |  | |
|  | When your child is supposed to be focusing on something else (e.g., studying) | | 5 | | 18 | | 24 | | 66 | | 290 | |  | |
|  |  | | 1.2% | | 4.5% | | 6.0% | | 16.4% | | 72.0% | |  | |
|  | At bedtime | | 5 | | 26 | | 27 | | 85 | | 260 | |  | |
|  |  |  | 1.2% | | 6.5% | | 6.7% | | 21.1% | | 64.5% | |  | |
|  |  | |  | |  | |  | |  | |  | |  | |
|  | While waiting (e.g., at bus stop) | | 139 | | 146 | | 30 | | 71 | | 17 | |  | |
|  |  |  | 34.5% | | 36.2% | | 7.4% | | 17.6% | | 4.2% | |  | |
|  |  | |  | |  | |  | |  | |  | |  | |
|  | In the bathroom (toilet) | | 75 | | 96 | | 85 | | 96 | | 51 | |  | |
|  |  |  | 18.6% | | 23.8% | | 21.1% | | 23.8% | | 12.7% | |  | |
