# Supplementary material for: Parental views on their children’s smartphone use during personal and relational activities
Source: PLoS One. 2024 Aug 5;19(8):e0308258. doi: 10.1371/journal.pone.0308258 (PMC11299814; doi:10.1371/journal.pone.0308258)
Supplement: Attachment — Submitted filename: Response to Reviewers.docx [file pone.0308258.s004.docx]

**Response to reviewers**

Dear Editor, dear Reviewers,

We would like to thank the Editor and the Reviewers for their valuable comments on our manuscript.

We believe that we have addressed all key concerns while preserving the scientific aims of the original submission. It is our opinion that the revised manuscript has improved in terms of quality and clarity.

A detailed item-by-item responses to all comments follow.

We have addressed all the additional Journal Requirements, incl. data availability and funding information.

**Reviewer #1:**

The paper "Parental views on their children’s smartphone use during personal and family routines" examines parental views on children's smartphone use during their daily routines and children's SU during these routines. The manuscript is well written, and the analyses are appropriate, but I have some issues with the terminology and the construct/definition of these routines.

#1) For me, daily routines represent some mundane and "automatic" activities, such as brushing teeth, so I was confused when I read your manuscript. When looking into your items, they seem to be family/peer interactions or activities, not necessarily routines. I would recommend renaming these routines in such a way or providing a more clear explanation in the introduction of how and why these interactions and activities are considered routines. I would also recommend dividing routines into categories of family and personal routines, as parents may have different views on these two categories and children may behave differently. Following this, I think abbreviations in the text would benefit from this (e.g., SUPR: personal routines, SUFR: family routines).

***Response*:** Thank you for your comment! We initially thought 'activity' was too general and chose 'routines' to emphasize the frequency and repetition of these activities. However, we now agree that 'activity' is more appropriate. We changed smartphone use during routines (SUR) to smartphone use during personal and relational activities (SUPRA). We specified more this while introducing the variable. For each SUPRA item we also specified whether it concerns personal or interpersonal/family activity. When reporting, we first reported all relational SUPRA and then all personal SUPRA items for higher clarity.

#2) As I mentioned, some of the routines are related to interactions with family members or peers, and you look into how they are related to the SU. This can be seen as technoference and/or phubbing, and I think your paper would benefit from discussing your results in this context, at least for the routines that are related to the interaction with others.

***Response*:** Thank you for this suggestion. In the introduction, we clarified that the use of smartphones during interpersonal interactions has been previously studied under terms technoference/phubbing:

*“For instance, smartphone use (SU) during interpersonal interactions, which has been previously studied under terms “technoference” or “phubbing”, seems to be associated with lower relationship satisfaction between friends [9] as well as between romantic partners [10–13]. In the professional context, smartphone use during face-to-face negotiation led negotiators to appear less trustworthy and less professional [14]. Regarding family life, parental use during parent–child interactions (parental technoference) has been linked to decreased family satisfaction [15], suboptimal parent–child interactions, children’s externalising and internalising behavioural problems [12], and parental inattentiveness to children’s safety and emotional needs [16]. SU during interpersonal interactions has been predominantly studied among adults, but the occurrence of occasional (42%) or frequent (30%) distraction by smartphones has also been reported for adolescent children during face-to-face conversations with their parents [17].”*

#3) All in all, this paper is novel in the field and shows some new perspectives on children's SU, but would benefit from more clear construct definition and clarification.

***Response*:**

We clarified more the process how SUPRA was constructed:

*“Fourteen personal, interpersonal and family activities during which a smartphone can be used were proposed. Eight activities were derived from the scientific literature cited above, which suggested that smartphone use during these activities might be harmful:*

- When your child is supposed to be focusing on something else (e.g., studying) [Personal activity]
- At bedtime [Personal activity]
- During a conversation with a peer (e.g., sibling, friend) [Relational activity]
- When playing with a peer (e.g., sibling, friend) [Relational activity]
- When you are saying something important to your child [Relational activity]
- During a parent‒child conversation [Relational activity]
- During a family mealtime [Relational activity]
- During dinning in the restaurant [Relational activity]

*Six additional activities were included to achieve greater variety in both personal and family activities:*

- While waiting (e.g., at bus stop) [Personal activity]
- In the bathroom (toilet) [Personal activity]
- When visiting family friends [Relational activity]
- When walking or hiking or being on trip together [Relational activity]
- When attending a cultural performance (e.g., theatre, cinema, concert) [Relational activity]
- When travelling/commuting together [Relational activity]

We believe that this also helped us to better convey the message why to focus on smartphone use during personal and relational activities.

#4) I would recommend not using abbreviations in the first sentence of the abstract. It would be better to write down the whole expression and put the abbreviation in the brackets (as is in the main text).

***Response*:** Thank you, changed accordingly.

#5) When describing scales of parental warmth and control, use the full name of the scale when first mentioning it.

***Response*:** Thank you for your very appropriate suggestion. We have now included the full names of the scales when first mentioning the scales of parental warmth and control.

#6) There are also several citing issues and errors I saw throughout the manuscript; read and revise these errors carefully.

***Response*:** Thank you, corrected.

-----------

**Reviewer #2:**

This study uses a sample of parents of children (aged 6-18) from Czech Republic to quantitatively describe parents’ perceptions of children’s use of smartphones during some routine events. The results reveal differences based on children’s age and some interesting associations with parents’ attitudes and parental warmth and control. Below are some of my suggestions for improvement:

1. In the abstract, it might be helpful to mention the age range and gender of both the parent sample and of their children.

***Response*:** Thank you. We have now included the age range and gender of both the parent sample and their children in the abstract.

2. Page 4, “Guidelines for parental regulation of specifically SU have been proposed by the Italian Paediatric Society,…”: What about from other organizations such as the American Academy of Pediatrics?

***Response*:** Thank you for this comment. We reported guidelines proposed by Bozzola et al. (2019), because they specifically mentioned smartphone use (and its possible collision with important personal activities). In contrast, the AAP guidelines (COUNCIL ON COMMUNICATIONS AND MEDIA, 2016), while more widely recognized, , were older and rather general. However, as the updated AAP guidelines from April 2024 (American Academy of Pediatrics, 2024) addressed specifically smartphone use, we added them to the text:

“*American Academy of Pediatrics (AAP) regularly publishes recommendations for parents concerning the regulation of screen media in general, with the newest update in April 2024 [26]. AAP guidelines are probably the most widely recognized. Although they are relatively general concerning screen media use, some parts are specifically relevant to smartphone use, particularly the fourth component called 'Crowding Out,' which prompts parents to ask: 'What does media get in the way of?'. For parents of children with their own smartphone it is recommend to prompt children to restrain from device use during school, homework, bedtimes [31]*. *Similar guidelines have been proposed by the Italian Paediatric Society [1], recommending parents to restrict their children from using smartphones during mealtimes, bedtimes, and homework.”*

We are also aware that other societies (e.g., Canadian Paediatric Society, Institute de France Académie des Sciences, British Royal College for Pediatrics and Child Health) developed guidelines on screen media use, but as our focus was on smartphone use, which is only marginally mentioned or not addressed in these guidelines, we chose not to report on them in detail..

3. “this relationship could not be fully attributed to the cross-sectional design of synthesised studies [27].” I have a hard time understanding this sentence. Please clarify more.

**Response:** Thank you for your feedback. We have divided and rewritten the sentence for better clarity:

*“In older adolescents, restrictive parenting was positively and moderately associated with increased problematic online screen media use (the pooled correlation was found to be .25 [.05, .44]) [29]. This positive correlation between increased restrictive parenting and problematic media use could be partially explained by cross-sectional design of most studies, while it makes sense that when problematic use emerges, the restrictive activity of parents increases [29]. However, a longitudinal study showed that the association was present even when the analysis was controlled for previous problematic use – i.e., problematic use led to increased restrictions but the restrictions further promoted problematic use in the next wave [31].”*

4. Page 5: This is a really long paragraph. Please consider breaking it down.

**Response:** Thank you for your suggestion. We have reviewed the paragraph and have broken it down into shorter sections for better readability.

5. Overall, in the introduction, there is a lack of in-depth literature review on children’s SUR and related phenomena, such as smartphone use during dinner time and during time spent with family. And there is a lack of literature review on parents’ perceptions of this.

**Response:** We conducted a thorough literature search but identified only a small number of directly relevant sources. However, we provided a review on a closely related topic—parental views on screen media in general. In total, we have added 10 new sources.

6. A concerning part of the method is that the selection and definition of the 14 routines being studied are unclear and seem arbitrary. Please explain the scientific method/procedure for how these 14 routines were determined.

***Response*:** Some routines (or activities as we renamed according to reviewer’s 1 suggestion) were derived from scientific literature, particularly those personal, interpersonal, and family activities during which smartphone use might be harmful; namely bedtime, bathroom time, activities requiring attentional focus – learning, interacting with peers, parent-child interactions, family mealtimes. We built upon this knowledge to derive 8 activities:

Personal activities (2 items)

- When he or she is supposed to be focusing on something else (e.g., studying)
- At bedtime

Interpersonal activities (either peer-to-peer or parent-child) (4 items)

- During a conversation with a peer (e.g., sibling, friend)
- When playing with a peer (e.g., sibling, friend)
- When you are saying something important to your child
- During a parent‒child conversation

Family activities (2 items)

- During a family mealtime
- During dinning in the restaurant

Additionally, we included other relevant activities identified in a qualitative study (Hrabec et al., manuscript in preparation) recently conducted with parents, which focused on their children's smartphone use.

Personal activities (2 more items)

- While waiting (e.g., at bus stop)
- In the bathroom (toilet)

Family activities (4 more activities)

- When visiting family friends
- When walking or hiking or being on trip together
- When attending a cultural performance (e.g., theatre, cinema, concert)
- When travelling/commuting together

We clarified this in the manuscript. Please refer to the Measure section for the detailed description.

7. Some of the routines are quite confusing. For example, what does "during a visit" mean/?

***Response*:** Thank you very much for pointing this out. The original Czech item in full was 'When visiting family friends,' meaning the situation where the whole family (or a parent with a child) meets family friends. This was the context we asked parents to report about. We have now included the entire item for clarity.

8. For between-group comparison, why choosing 10/11 to be the breaking point for children versus adolescents?

***Response*:** The choice of 10/11 as the breaking point between children and adolescents was based on the presumption that significant changes in screen media use occur around this age. This is also reflected in different guidelines for children younger than 10 years and older, such as those from the American Academy of Pediatrics (2024). We have clarified this in the Methods section when reporting the statistical analysis:

*Analyses were conducted separately for parents of children (6–10 years old) and parents of adolescents (11–18 years old). Two categories were formed based on the presumed differences in the screen media use of (and the recommended parenting strategies for) children younger and older than 10 years (American Academy of Pediatrics, 2024).*

9. I think results from the regression model should drive the main result interpretation and discussion rather than the correlations. In the regression model, it is important that many potential confounding factors, including the sociodemographic characteristics of both parents and children, are controlled.

***Response*:** Thank you for this comment. We acknowledge the limits of relying on correlations while the more advanced statistical test (regression analysis) is available. On the other hand, the study was designed as explorative and the discussion was therefore driven mostly by descriptive results (e.g., the average dislike of parents for various SUPRA, the frequency of various SUPRA).

However, we have taken your valuable comment into account and have reformulated the relevant paragraph in the discussion section to clarify clear that the parental predictors of SUPRA dislike were identified using linear regression analysis:

*“Less positive attitudes towards screen media, higher parental warmth and control and being female (mother) were all found to significantly contribute to high parental dislike of SUR even when analyzed together using regression analysis. Positive parental attitudes towards screen media have been previously studied mostly as a factor contributing to…”*

We also replaced the table with the results of regression analysis from the supplement to the main body (Table 4).

In line with this comment, we have removed two paragraphs from the Results section.

1) Second saying that SUPRA was positively associated with daily smartphone time and negatively associated with parental warmth, with a positive correlation between SUPRA and PATS in children but a negative correlation in adolescents.

2) Second saying that Smartphone ownership was the only sociodemographic factor significantly associated with children's SUPRA, with notable differences in usage patterns between owners and nonowners, though no significant differences were found in the total number of routines involving frequent smartphone use.

---------------

**Additional changes** that were based on / stemmed from the Reviewers’ comments in the manuscript

Thus, we have adjusted the accordingly to the comments:

- Abbreviation SUPRA (smartphone use during personal and relational activities) is now being used throughout the manuscript and in the Tables.
- Title to better fit the content, using the term: *relational activities*.
- Discussion section (notably the first paragraph regarding aims and the second paragraph the most negatively perceived SU by parents).
- Conclusions.
- Table 1 and Table 2 (were restructured).
- Supplementary Tables 2 and 3.

There were also minor linguistic adjustments done in the manuscript to improve readability and clarity of the text. Please, refer to the document with changes tracked.
