# Supplementary material for: Parental views on their children’s smartphone use during personal and relational activities
Source: PLoS One. 2024 Aug 5;19(8):e0308258. doi: 10.1371/journal.pone.0308258 (PMC11299814; doi:10.1371/journal.pone.0308258)
Supplement: Attachment — Submitted filename: PlosISU_Revision2_Response2reviews_fin.docx [file pone.0308258.s005.docx]

**Response to reviewers**

Dear Editor, dear Reviewers,

We would like to thank you for acknowledging changes we made in the manuscript and one again for your constructive and useful feedback.

We believe we have addressed additional issues raised (by Reviewer #1) that resulted, in our belief, in the increased quality of the manuscript.

A detailed item-by-item responses to all comments follow:

**Reviewer #1: I** thank the authors for the revised manuscript. The ideas of the manuscript are now more clear, but with this clarification of the main goal of the manuscript, I found the manuscript to be a bit confusing.

In the abstract, you present your goals as follows: "This study aimed to (i) assess parental dislike (PD) of SUPRA, (ii) estimate SUPRA frequency, and (iii) identify predictors of PD of SUPRA" but on page 7, you put frequency of SUPRA as the last goal, and there is also confusion with the presentation of the result. It would benefit your paper, in terms of being concise and easier to read, to present your goals from a wider range (i.e., frequency of SUPRA) to a specific one (i.e., parental views/dislikes of SUPRA). So I recommend to authors that they think about rearranging the goals of the manuscript in such a way as to get a clearer picture of their study and study goals.

**Response:** Thank you for raising this point.

We have rearranged the aims in the Abstract as suggested – i.e., from (i) SUPRA frequency to (ii) parental dislike of SUPRA magnitude and (iii) its predictors. We rearranged methods, results and discussion accordingly to mirror this logic.

**Reviewer #1:** Following that, the discussion of the paper lacks a theoretical explanation of the obtained results. For example, in the beginning of the discussion, you start with "The most negatively perceived was SU during two relational activities: when attending a cultural performance and when a parent is trying to say something important to their child. And additionally, SU during two personal activities: during bedtime and when a child is supposed to focus on something else, such as studying. In contrast, parents were generally okay with two SUPRA: (during waiting and during travelling/commuting), suggesting that SU during these activities may not be seen as intrusive by most parents." However, I haven't seen, throughout the discussion, any explanation as to why you think you got these results. For relational activities, it could be that parents perceive these activities as a chance to bond with children or spend quality time together, and for personal activities, parents may think about the consequences for a child's development, well-being, school achievement, etc. I am certain that there are some theoretical explanations for these and other results you obtained, so I encourage you to include them in your manuscript, as it would give more support to your findings.

**Response:** Thank you for bringing this to our attention. Given that we have reorganized the order in which we presented aims and reported results, we also reorganized discussion, starting with frequency of SUPRA. Each paragraph on frequency of SUPRA was then supplied with the results on parental dislike of this particular SUPRA, which (as we believe) improved the overall readability of discussion. We included additional sources to better discuss the high dislike of parents for most SUPRA (in total, six new sources on parental attitudes toward screen media were added, mostly reports of qualitative studies). We believe that our discussion of the results has been significantly improved.

**Reviewer #1:** I have some other comments and suggestions:

- I recommend including some keywords that reflect your focus on personal and relational activities and maybe excluding the "family" keyword.

**Response:** Thank you, we added “smartphone use during personal activities” and “technoference” and “phubbing” as keywords for smartphone use during relational activites

**Reviewer #1:** - On page 4, in the sentence "This may be due to perceived differences in the purposes of technology use, while daughters are believed to use it predominantly for social networking and sons for gaming [27]" it seems that the word "while" should be exchanged with the word "where".

**Response:** Done

**Reviewer #1:** - On pages 5 and 6, you mention problematic screen media use, but you never define this term.

**Response:** Done *(“...problematic screen media use [33–35], which can be defined as excessive media engagement with impaired control over use and with negative consequences in major areas of life functioning [36]“)*

**Reviewer #1:** - Descriptions of Parental warmth and Parental control are missing the used measurement scale.

**Response:** Thank you, added.

**Reviewer #1:** - "Characteristics" should be included in the method under the Participants section.

**Response:** Included, thank you.

**Reviewer #1:** - Before the results of the regression analysis, put a heading, such as "Predictors of Parental Dislike of SUPRA" or something similar.

**Response:** Done accordingly.

**Reviewer #1:** - Have you controlled for other sociodemographic variables in the regression model? Whether the answer is yes or no, you should clarify this in the results (under the regression table), i.e., explain why you have or haven't included other variables.

**Response:** We added the information into Methods (“*These predictors were selected based on whether they were found to be significantly associated with PD of SUPRA, i.e., on previous step.”)* and also when reporting the results: *“Using the principle of parsimony, we included only variables significantly associated with the PD of SUPRA in the model.”*

**Reviewer #1:** Overall, the paper shows great improvement after the revision, and I thank the authors for considering the previous comments. The paper describes something still relatively unknown in the literature, and as such, it would benefit from a more theoretical background.

**Response**: The literature issue has been addressed. Thank you for your time invested in our work.
